# Identification of genes and variants associated with quantitative traits using Bayesian factor screening

**DOI:** 10.1186/1753-6561-5-S9-S4

**Published:** 2011-11-29

**Authors:** Kith Pradhan, Seungtai Chris Yoon, Tao Wang, Kenny Ye

**Affiliations:** 1 Department of Epidemiology and Population Health, Albert Einstein College of Medicine, 1300 Morris Park Avenue, Bronx, NY 10461, USA; 2 Seaver Autism Center, Department of Psychiatry, Mount Sinai School of Medicine, Box 1668, One Gustave L. Levy Place, New York, NY 10029, USA

## Abstract

We propose a factor-screening method based on a Bayesian model selection framework and apply it to Genetic Analysis Workshop 17 simulated data with unrelated individuals to identify genes and SNP variants associated with the quantitative trait Q1. A Metropolis-Hasting algorithm is implemented to generate a posterior distribution in a restricted model space and thus the marginal posterior distribution of each variant. Our framework provides flexibility to make inferences on either individual variants or genes. We obtained results for 10 simulated data sets. Our methods are able to identify *FTP1* and *KDR*, two genes that are associated with Q1 in a majority of replicates.

## Background

Yoon [1] proposed a Bayesian framework for factor screening, based on Bayesian model selection. In summary, given a total of *p* candidate factors, we evaluate a model space of *K* models. Each model involves *m* factors (usually *m* is much smaller than *p* for the Pareto principle of factor scarcity and is specified by the user), is of the same class, and is assigned a prior probability *p*(*M_k_*). The prior probabilities are usually the same if the models have the same number of parameters. Note that a model *M_k_* corresponds to a binary vector *γ* = (*γ*_1_, *γ*_2_, …, *γ_p_*), where . Given observed data *y*, the posterior probabilities are given by:(1)

in which *p*(*y* | *M_k_*) is obtained by integrating out the unknown parameters. To evaluate the importance of each factor, we can summarize its marginal posterior probability by:(2)

Here, we apply this method to the Genetic Analysis Workshop 17 (GAW17) simulated data, of which the genotypes of 697 individuals at 3,205 genes are based on exome sequencing data of the 1000 Genomes Project [2]. In principle, the general framework described applies to any class of parametric or nonparametric models, so long as the model posterior probability is well defined and can be computed. However, here we consider only linear models, and we apply our method to analyze quantitative trait Q1 of the GAW17 data set. This trait was simulated to be associated with 39 single-nucleotide polymorphisms (SNPs) in 9 genes. In the first part of our analysis, we treat each SNP as a factor to evaluate the association at the SNP level. In the second part of our analysis, we treat each gene as a factor and evaluate the association at the gene level. Because each gene contains a different number of SNPs, unequal prior probabilities are assigned to candidate models to penalize models with more parameters based on the Bayesian information criterion (BIC) [3].

## Methods

### Use SNPs as factors

Consider a linear model with quantitative responses *y* = *Xβ* + *ε*, where *y* is a quantitative trait and *X* is a matrix derived from genotypes from *m* SNP variants, with details to be given later, and *ε* ~ *N*(0, *σ*^2^*I*). The prior distribution of *β* and *σ*^2^ is given as *β* | *σ*^2^ ~ *N* (0, *σ*^2^ (*λ*^2^Σ)) and *p*(*σ*^2^) ∝ 1/*σ*^2^, where *λ* is a tuning parameter that controls the flatness of the prior distribution and *Σ* can be specified to reflect the relation between the candidate variables. In this notation, we omit the subscript vector *γ* that represents each model *M_k_* in the model space. Under these prior distributions, the posterior distribution of *γ* can be easily computed by integrating out *β* and *σ*^2^:(3)

where . Brown and Vannuci [4] developed a method that further improves the computational efficiency of the posterior distribution, because it can be written as:(4)

where:(5)(6)

Note that  is the sum residual squared when we regress ỹ on ; it can be computed as , and the other part of the posterior distribution can be computed as , where  is the QR decomposition of . The details of using QR decomposition for least-squares estimates of linear models are given by Seber [5].

However, even with efficient evaluation of the model posterior distribution, the model space contains *p* choose *m* models and is usually too big to be exhaustively evaluated. For example, with *p* = 24,478 variants, there are almost 2.5 trillion models when *m* = 3. If 1 million models are evaluated in a second, it will take almost a month to evaluate all 2.5 trillion models. Therefore a Monte Carlo Markov chain (MCMC) method is used to obtain the posterior probability. We used a simple Metropolis-Hasting algorithm, which we briefly described in what follows. Let *γ*^(^*^j^*^)^ be the model at the *j*th step. At the (*j* + 1)th step, a model γ* is randomly selected by replacing a random active factor (i.e., *γ_i_*^(^*^j^*^)^ = 1) in *γ*^(^*^j^*^)^ with a random inactive factor (i.e., *γ_i_*^(^*^j^*^)^ = 0) in *γ*^(^*^j^*^)^. Set *γ*^(^*^j^*^+1)^ = γ* with probability min{*p*(γ* | *y*) / *p*(*γ*^(^*^j^*^)^ | *y*),1}; otherwise, set *γ*^(^*^j^*^+1)^ = *γ*^(^*^j^*^)^. To estimate the marginal posterior distribution of each factor, we simply count the number of times the factor appears in the chain. To determine if the MCMCs have reached convergence, we usually compare two chains run with different seeds given to the pseudo–random number generator.

Given a vector of active factors (genotypes variants) *γ*, each column of *X* is the number of minor alleles of each (active) variant in the 697 subjects, centralized to mean 0. Note that all models with a fixed number of active factors have the same number of parameters. This avoids the complication of comparing models with different numbers of unknown parameters; thus all candidate models have the same prior distribution. Our framework can consider factor-factor interactions by including interaction terms in the model matrix *X*, but we do not include them here.

The effects of the three covariates, Smoke, Age, and Sex, are removed by taking a regression of the quantitative traits to the covariates. The residuals are then used as the quantitative response to be associated with genetic variants. Because Q1 is simulated with interaction effects between variants in the *KDR* gene and smoking, by ignoring such interactions, our power for detecting the *KDR* gene would be lower.

### Use genes as factors

Using SNP variants as factors does not take into consideration genes on which variants are sitting. Statistical inference on a gene can be made indirectly by averaging the marginal posterior distribution of all variants within it. Alternatively, we can treat each gene as a factor in our factor screen framework by forcing all variants in a gene in and out of a linear regression model together. The inferences are then made directly on each gene, but no inference is made on individual SNPs.

More specifically, in the linear model *y* = *Xβ* + *ε*, *X* is a matrix derived from genotypes of all SNP variants of *m* “active” genes. The number of columns of *X* is the total number of SNP variants of the *m* genes. A binary vector *γ* = (*γ*_1_, *γ*_2_, …, *γ_p_*) now represents the active or inactive state of the 3,205 genes, but the computation of the posterior distribution follows the same formula as above. However, each gene has a different number of SNP variants; hence the column of *X* varies even though the number of active genes is fixed. Therefore the total number of parameters of each candidate model is not fixed. If we still assign each candidate model with equal prior probability, the model selection procedure tends to bias toward genes with a greater number of variants. In an effort to correct for such bias, we assign prior probabilities of candidate models based on the BIC. That is, the prior probability of a model is proportional to *e*^–^*^k^*^/2^, where *k* is the total number of parameters.

## Results

Running a MCMC procedure is computationally expensive. Therefore we run our analysis on only the first 10 replicates of the 200 replicates simulated by the GAW17 data set. However, we believe that the results of these 10 replicates are sufficient to evaluate our method.

### Use SNP as factors

**Figure 1:**
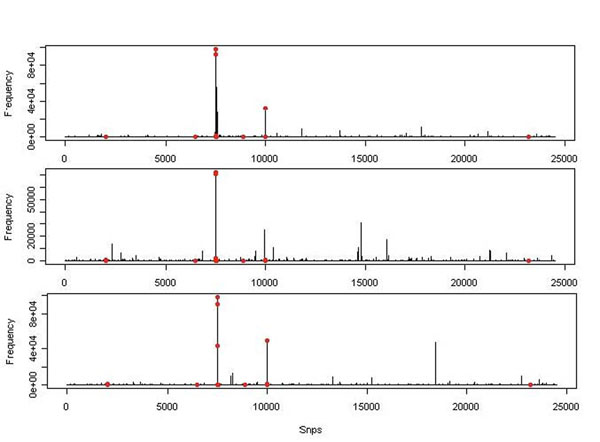
**Marginal posterior probability of SNP variants** The marginal posterior probability of 24,487 variants obtained for the first three replicates are displayed. Red dots mark the Q1-associated variants.

We analyzed Q1 with *m* = 10. That is, our model space contains all models with 10 active SNP variants. In the prior distribution of *β*, we set *λ* = 1 as the effects of the Q1-associated variants in the simulation range from 0.13 to 1.35. We also set *Σ* as an identity matrix to reflect no prior information on how effects of those variants are associated. We ran a MCMC of length 100,000 (after a burn-in run of 1,000) on each replicate and computed the marginal posterior probabilities. To check whether the length of the MCMC was good enough, we ran another independent MCMC of length 100,000 on the first replicate. The marginal posterior probabilities estimated from the two chains were highly correlated, suggesting convergence at such a length. The marginal posterior probability of the first three replicates is illustrated in Figure 1. The red dots represent SNPs associated with Q1. From Figure 1, one can see that several Q1-associated SNPs consistently have high posterior probabilities, but most have low posterior probabilities and are not detected. In addition, in each replicate, some SNPs that are not associated with Q1 also have high posterior probabilities, which could lead to false positives, but such false positives are largely not repeated in the replicates.

The purpose of factor screening is to select a set of factors for further investigations. Therefore a certain level of false positives can be tolerated. There are in general two ways to make a cutoff. One is to take a certain number of factors from the analysis; the other is to take factors with a certain level of marginal posterior probability. Table 1 shows the list of variants that ever appeared among the top 20 SNPs in 10 replicates and the number of times they appeared. Four SNPs that appeared most frequently were all Q1 SNPs. They are C13S523, C13S522, and C13S524 of *FLT1* and C4S1884 of *KDR*. Ten variants appeared twice in 10 replicates, among which only two are Q1 associated. One hundred fifty-three variants appeared only once, among which only three are Q1 associated. Out of the top 20, we have on average 3.3 Q1 variants per replicate. Overall, 30 of 39 Q1-associated variants never appeared in the top 20 in any of the 10 replicates. However, among the seven Q1 variants with a minor allele frequency (MAF) greater than 0.01, only one (C14S1734 of *HIF1A*) with an MAF of 0.012195 was never detected in 10 replicates. Among nine Q1 variants that appeared in the top 20, C6S22981 (*VEGFA*) has the least MAF, 0.002152, corresponding to three alleles among the 697 subjects. Table 2 lists seven Q1 SNP variants with marginal posterior probabilities larger than 0.1 in any of the 10 replicates, all of which are also listed in Table 1. There are only 3 occurrences of non-Q1 SNPs with a posterior probability greater than 0.5 and 36 occurrences with a posterior probability greater than 0.1. That is, only 0.3 and 3.6 per replicate.

**Table 1:** SNPs with high marginal posterior probabilities

Appearances in top 20 SNPs among 10 replicates	SNPs (genes), *m*=10
9	C13S523 (*FLT1*)
8	C13S522 (*FLT1*)
5	C13S431 (*FLT1*)
4	C4S1884 (*KDR*)
2	C4S1878 (*KDR*), C6S2981 (*VEGFA*), 8 SNPs *not* associated with Q1
1	C13S524 (*FLT1*), C1S6533 (*ARNT*), C4S1890 (*KDR*), 151 SNPs *not* associated with Q1

The SNP variants that have posterior probabilities in the top 20, including both Q1-associated SNPs and false positives, are listed. The symbols of Q1-associated variants are given and those not associated with Q1 are omitted. The first column shows the frequency of a variant in the top 20 among 10 replicates.

**Table 2:** SNPs with high marginal posterior probabilities

SNP	Gene	Posterior probability > 0.5	Posterior probability > 0.1
C13S431	*FLT1*	1	4
C13S522	*FLT1*	6	8
C13S523	*FLT1*	9	9
C13S534	*FLT1*	0	1
C4S1878	*KDR*	0	2
C4S1884	*KDR*	2	4
C6S2981	*VEGFA*	0	1
Other Q1		0	0
Non-Q1		3	36
False-positive discovery rate		3/21	36/65

The first column lists Q1-associated SNP variants that have marginal posterior probabilities larger that 0.1 in any of the 10 replicates. The second column gives the genes on which these variants sit. The third and fourth columns are frequency of their posterior probabilities larger than 0.1 and 0.5, respectively; they summarize all variants *not* associated with Q1 but with a posterior probability greater than 0.1 or 0.5. The false discovery rates are given in the last row.

From the results of the analysis at the SNP level, it is also possible to make inferences about the genes by computing the average marginal posterior probability per SNP for the genes. Table 3 shows the ratio between this average marginal posterior probability of the nine Q1-associated genes to the prior probability (which equals 10/24,487). Six out of nine genes had a posterior probability greater than the prior probability, with *FLT1* and *KDR* on average 113 times and 50 times greater, respectively.

**Table 3:** Average marginal posterior/prior probability ratio per variants for Q1-associated genes

Gene	Posterior/prior ratio (mean of 10 replicates)
*ARBT*	1.79
*ELAVL4*	1.31
*FLT1*	113
*FLT4*	0.649
*HIF1A*	0.970
*H1F3A*	0.483
*KDR*	50.5
*VEGFA*	11.6
*VEGFC*	2.50

For each of the Q1-associated genes, the average marginal posterior probability over all its SNP variants is computed and divided by the average marginal prior probability 10/24,487. The second column lists such ratios averaged over 10 replicates. A higher ratio indicates more importance of the gene.

### Use genes as factors

For the first 10 replicates, we analyze Q1 with *m* = 3 and *m* = 6. We run 10,000 iterations of MCMCs after a burn-in period of 1,000. A comparison between the two independent chains for the first replicate suggests that the marginal posterior probabilities of the individual factors converge at such length. For the prior probability of *β*, we set *λ* = 1, as previously, and set *Σ* as an identity matrix. We also run our analysis with a *Σ* that imposes a slight correlation (at 0.1) among the effects of variants within a gene. The results are similar and hence are not presented here.

Similar to Table 1, Table 4 lists the genes that ranked in the top 10 marginal posterior probabilities in any of the 10 replicates. We can see that the results at *m* = 3 and *m* = 6 are similar. In fact, *FLT1* always had the highest marginal posterior probability in all 10 replicates. At *m* = 3, *KDR* appeared five times and *HIF1A* and *ARNT* appeared once. Eighty-one genes not associated with Q1 also appeared, with 79 appearing only once and 2 twice. At *m* = 6, *KDR* appeared six times, and *HIF1A* and *VEGFA* appeared once. Seventy-eight genes not associated with Q1 also appeared, with 74 appearing once and 4 twice. To see whether or not our BIC-based adjustment of the prior probability was fair, we compared the number of SNPs of those 78 genes with the other 3,118 genes that are not Q1 associated. The number of SNPs per gene is much lower among the 78 genes (3.45) than among the rest of the genes not associated with Q1 (7.73). This suggests that our prior probability assignment overcorrects and favors genes that have fewer variants. One reason for this could be high correlation between the variants and singularity of some model matrices, making the effective number of parameters less than the number of variants in the model. Better prior probability assignment will be further investigated.

**Table 4:** Genes with high marginal posterior probabilities

Appearances in the top 10 genes among 10 replicates	*m* = 3	*m* = 6
10	*FLT1*	*FLT1*
6	−	*KDR*
5	*KDR*	−
2	Two genes *not* associated with Q1	Four genes *not* associated with Q1
1	*ARNT*, *HIF1A*, 79 genes *not* associated with Q1	*HIF1A*, *VEGFA*, 74 genes *not* associated with Q1

The genes that have posterior probabilities in the top 10, including both Q1-associated genes and false positives, are listed. The symbols of Q1-associated genes are given and those *not* associated with Q1 are omitted. The first column shows the frequency of a variant in the top 10 among 10 replicates. The second and third columns show the results at *m* = 3 and *m* = 6, respectively, where *m* is the fixed number of factors entertained in a model of the model space.

Table 5 shows the marginal posterior probabilities of the nine Q1-associated genes averaged over 10 replicates and the number of times they are greater than 0.5 and 0.1. The marginal posterior probability for *FLT1* is almost 1 in every replicate. *KDR* has an average posterior probability greater than 0.32 when *m* = 3 and about 0.4 when *m* = 6. *VEGFA* is the only other gene that ever has a marginal posterior probability greater than 0.1, which occurs once in 10 replicates. But the number of false positives is also low. At *m* = 3, the false-positive discovery rate is 5/19 and 18/32 at the 0.5 and 0.1 cutoffs, respectively. At *m* = 6, higher false-positive discovery rates are observed at 7/20 and 51/68. We also observe that the posterior probability of *ARBT* is somewhat higher at *m* = 3 than at *m* = 6. This could be explained by *ARBT* having a large number of variants (18 SNPs), and our procedure penalizes genes with many variants more harshly at *m* = 6 than at *m* = 3. For the same reason, *VEGFA* (6 SNPs) and *VEGFC* (1 SNP) fair better at *m* = 3 than at *m* = 6.

**Table 5:** Marginal posterior probability for Q1-associated genes

Gene	Posterior probability (mean of 10 replicates)		Posterior probability > 0.5		Posterior probability > 0.1	
	
	*m* = 3	*m* = 6	*m* = 3	*m* = 6	*m* = 3	*m* = 6
*ARBT*	0.00521	0.00175	0	0	0	0
*ELAVL4*	0.00001	0.00065	0	0	0	0
*FLT1*	0.98967	0.96252	10	10	10	10
*FLT4*	0	0.00053	0	0	0	0
*HIF1A*	0.00596	0.00704	0	0	0	0
*H1F3A*	0	0	0	0	0	0
*KDR*	0.32353	0.40953	4	3	4	6
*VEGFA*	0.0006	0.0434	0	0	0	1
*VEGFC*	0.00129	0.00256	0	0	0	0
Non-Q1			*5*	*7*	*18*	*51*
False-positive discovery rate			5/19	7/20	18/32	51/68

Marginal posterior probabilities of nine Q1-associated genes averaged over 10 replicates are listed in the second and third columns. For each gene, the number of times that its posterior probability is greater than 0.5 is listed in columns 4 and 5, and the number of times that it is greater than 0.1 is listed in columns 6 and 7. The second to the last row lists the total number of times that genes *not* associated with Q1 have a posterior probability greater than 0.1 and 0.5. The false-positive discovery rate is given in the last row. The results at *m* = 3 and *m* = 6 are listed in separate columns, where *m* is the fixed number of factors entertained in a model of the model space.

## Conclusions

We presented a simple method of Bayesian factor screening and applied it to analyze Q1, a quantitative trait simulated to be associated with 39 SNP variants in 9 genes. We applied two implementations: One treats each SNP as a factor; the other treats each gene as a factor. Our computational framework is simple, straightforward, and efficient. The prior probabilities require few assumptions and are as noninformative as possible. Based on our experience, the results are not sensitive to the choice of *λ* and *Σ* in the prior probability and are not sensitive to the choice of *m* when SNPs are treated as factors. No biological information was used, except in the second implementation, where we grouped the SNPs of the same gene together.

Our method is quite effective. In the gene-level analysis, when all SNPs in a gene are treated as a group, we are able to identify *FTL1* consistently as the top candidate and we find *KDR* about half of the time. Beyond these two genes, we are not able to identify other Q1 genes without dramatically increasing our false-positive discovery rate. Our prior probability assignment penalizes models with more parameters and tends to overly favor genes with fewer variants; it requires further adjustment. The false-positive discovery rate tends to increase as *m* increases. In the SNP-level analysis, we have a good chance of identifying most Q1-associated SNPs with MAF > 0.01, at a reasonable false-positive discovery rate. The rarer variants are difficult to identify, even allowing for a high false-positive discovery rate. It might be unreasonable to expect variants with a low MAF to be identified in a sample size of 697 and a low false-positive discovery rate when there are almost 25,000 candidate variants, unless some degree of biological knowledge is used. The inference on genes derived from SNP-level analysis seems reasonable effective, finding *FLT1* and *KDR* frequently. When we take a fixed number of top factors for further investigations, the false discovery rate tends to be high. However, multistage designs can be used to gradually weed out false positives that pass the early rounds, because the chance for them to luck out twice is very low.

## Competing interests

The authors declare that there are no competing interests.

## Authors’ contributions

KP and KY draft the manuscript. KP carried out the computational aspect of the work. SY helped in developing the algorithm and provided part of the codes. TW helped on the design of the study. KY conceived, designed and directed the study.
